# Feline gastrointestinal eosinophilic sclerosing fibroplasia in two cats: Serial ultrasonographic and computed tomography findings

**DOI:** 10.17221/2/2024-VETMED

**Published:** 2024-05-27

**Authors:** Daji Noh, Hyeeun Jo, Hyunguk Shin, Sang-Kwon Lee, Sooyoung Choi, Kija Lee

**Affiliations:** ^1^Department of Veterinary Medical Imaging, College of Veterinary Medicine, Kyungpook National University, Daegu, Republic of Korea; ^2^24 Africa Animal Medical Center, Daejeon, Republic of Korea; ^3^College of Veterinary Medicine, Kangwon National University, Chuncheon, Republic of Korea

**Keywords:** diagnostic imaging, FGESF, gastrointestinal tumor, inflammatory bowel disease

## Abstract

A 6-month-old Ragdoll and 9-year-old Russian Blue cat presented with vomiting. Ultrasonography and computed tomography showed a pyloric antrum mass with wall layering loss and regional lymphadenopathy in the Ragdoll kitten. The Russian Blue cat only presented with muscularis layer thickening throughout the jejunum; however, despite medications, it later progressed to a mass with wall layering loss on the serial ultrasound. Both cats underwent surgery, and feline gastrointestinal eosinophilic sclerosing fibroplasia (FGESF) was histologically confirmed. FGESF should be considered for gastrointestinal masses demonstrating wall layering loss and lymphadenopathy, even in kittens, and intestinal muscularis layer thickening that is refractory to medications.

Feline gastrointestinal eosinophilic sclerosing fibroplasia (FGESF) is a recently described eosinophilic nodular mass located within the gastrointestinal tract (Craig et al. 2009; Linton et al. 2015). Although a few studies and case reports have been reported on FGESF, computed tomography (CT) features are limited to the extramural retroperitoneal area and rectum (Thieme et al. 2019; Goffart et al. 2022). A recent study on FGESF reported ultrasonographic findings in 30 cats, revealing typical features such as a loss of intestinal layering, thickening, and mixed wall echogenicity with hyperechoic areas, particularly in gastric or intestinal cases (Cerna et al. 2024). However, there is a lack of thorough descriptions of serial ultrasonographic imaging changes in FGESF. Herein, we present two cases of FGESF and their clinical and diagnostic imaging features, including serial ultrasound and CT.

## Case description

**Figure 1:**
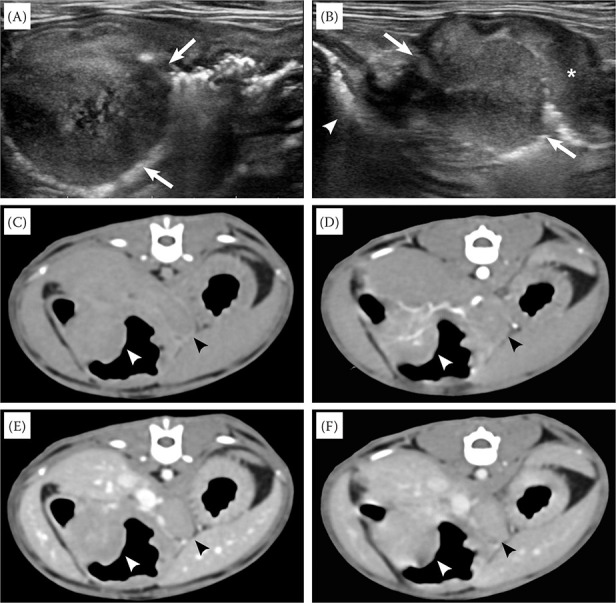
Ultrasonographic (A, B) and computed tomographic images, including the non-contrast (C), arterial (D), portal (E), and delayed (F) phases of the pyloric mass in case 1 (A) The pyloric mass (arrows) is heterogeneous with hyperechoic areas in the centre. It is pedunculated in shape with ill-defined margins and protrudes into the gastric lumen. (B) Normal intestinal layering of the ascending duodenum (arrowhead) and thickened gastric wall associated with loss of layering (asterisk) near the mass (arrows) are noted. (C–F) The mass at the dorsocaudal portion of the pyloric antrum protruding into the lumen (white arrowheads) with mild homogeneous contrast enhancement. An enlarged gastric lymph node is identified (black arrowheads)

A 6-month-old male vaccinated Ragdoll indoor kitten was referred to Kyungpook National University Veterinary Medical Teaching Hospital due to persistent vomiting since 2 months of age. The complete blood cell count, including eosinophils (0.8 × 10^9^/l; reference range 0.1–1.5 × 10^9^/l), and serum chemistry results were unremarkable. A soft tissue opacity mass in the pylorus region was noted on the abdominal radiography. A large, heterogeneously hyperechoic, intraluminal mass at the pyloroduodenal junction with a complete loss of wall layering was noted in the abdominal ultrasound (Prosound F75; Hitachi Aloka Medical System, Tokyo, Japan) (Figure 1A,B). This mass induced a severe outflow obstruction at the pyloroduodenal junction. The gastric wall adjacent to the mass was thickened with a loss of layering; however, no abnormalities were noted in the gastric body and fundus. Markedly enlarged pancreaticoduodenal and gastric lymph nodes were observed.

After air insufflation into the stomach to facilitate gastric wall identification, a thoracic and abdominal CT was performed using a 32 multislice CT scanner (AlexionTM; Cannon Medical System, Tokyo, Japan). Contrast studies were performed at 10, 30, and 90 s after a 600 mgI/kg iohexol injection (Omnipaque 300; GE Healthcare, Oslo, Norway). No abnormalities, such as enlarged lymph nodes or pulmonary nodules, were identified in the thoracic CT. A large, approximately 18** × **16** × **14 mm pedunculated soft tissue attenuating mass located dorsocaudal to the pyloric antrum was noted on the abdominal CT (Figure 1C–F). The mass showed a mildly diffuse homogeneous contrast enhancement with wall layering loss. The border between the thickened gastric wall and pyloric mass was not clearly distinguished. The gastric body and fundus were normal. Markedly enlarged gastric and pancreaticoduodenal lymph nodes with mild contrast enhancement surrounded by mild fat stranding signs were noted. No evidence of pulmonary nodules was observed. A primary gastric tumour, severe gastritis with hypertrophic pyloric gastropathy, feline infectious peritonitis, and FGESF were considered as the differential diagnoses based on the diagnostic imaging findings. Immediately after the CT scan, an ultrasound-guided fine-needle aspiration of the gastric lymph node was performed, and reactive hyperplasia was diagnosed. Famotidine (Gaster Tab; Donga ST, Seoul, Republic of Korea; 1 mg/kg q12 h), maropitant (Cerenia; Fareva Amboise, Porce sur Cisse, France; 1 mg/kg q24 h), and metronidazole (Flasinyl; HKinnoN, Republic of Korea; 15 mg/kg q12 h) were administered along with a prescription wet diet (Veterinary Diet Gastrointestinal; Royal Canin, Seoul, Republic of Korea) and the cat maintained good appetite with no vomiting symptoms.

**Figure 2:**
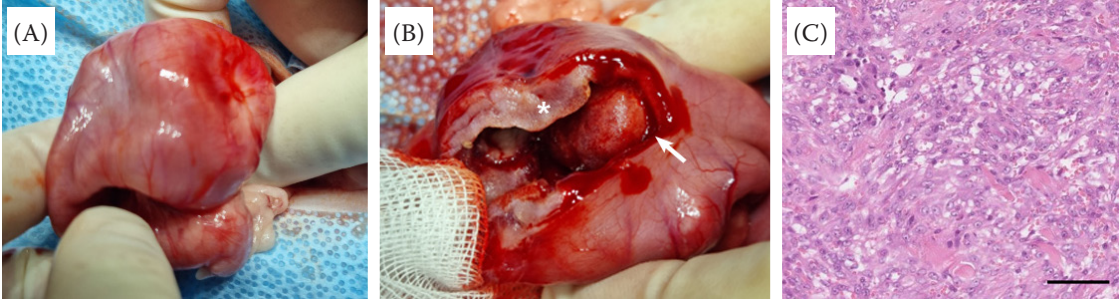
Macroscopic (A, B) and histopathological (C) photographs of the pyloric mass Macroscopic (A) and cross-sectional (B) photographs of the incised pyloroduodenal junction during surgical resection. Concentric thickened wall (asterisk) and firm intraluminal mass (arrow) are observed. Histology (C) of the resected lesion from the pyloric mass. Spindle cells are arranged in bundles and streams amid dense collagen bundles. Spindle cells have small amounts of eosinophilic cytoplasm and indistinct cell borders. Haematoxylin and eosin; scale bar = 50 μm

The pyloric mass was surgically removed a week after the CT, revealing a concentric thickened wall with a firmly pedunculated intraluminal mass at the pyloroduodenal junction. FGESF was histologically confirmed (Figure 2). Following the surgery, the patient developed postoperative acute pancreatitis with jaundice that showed no response to intensive medical treatment, as indicated by a strong positive SNAP feline pancreatic lipase test and an increased total bilirubin level (0.90 mmol/l; reference range, 0–0.05 mmol/l), and died 7 days later. No post-mortem examination was conducted.

A 9-year-old, intact female vaccinated Russian Blue indoor cat was presented to 24 Africa Animal Medical Center due to intermittent vomiting. The complete blood cell count, including eosinophils (0.24 × 10^9^/l; reference range 0.1–1.5 × 10^9^/l), and serum chemistry results were unremarkable and the abdominal radiographs were within normal limits.

**Figure 3:**
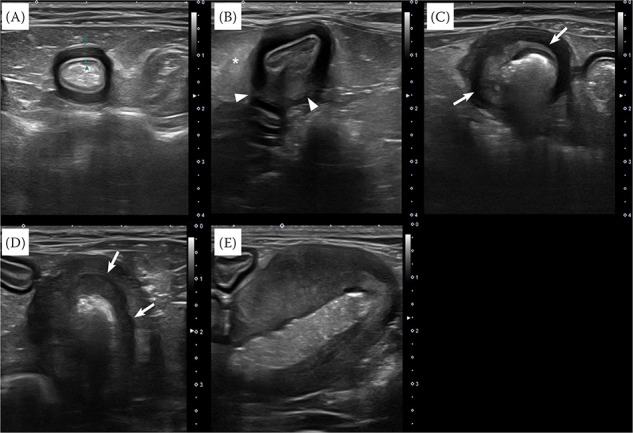
Serial ultrasonographic images of the jejunal mass at first presentation, 3, 5, 8, and 9 weeks (A–E) in case 2 (A) At first presentation, the muscularis layer is circumferentially thickened (full thickness, 3.5 mm; muscularis thickness, 2.0 mm) without apparent mass formation. (B) At 3 weeks, the same mass is poorly marginated, eccentric intramural, and shows altered layering (arrowheads). Increased muscularis layer thickness (full thickness, 4.3 mm; muscularis thickness, 3.1 mm) is identified surrounded by hyperechoic mesentery tissue (asterisk) at the same level. (C, D) Up to the 8^th^ week, the mass changes in shape from eccentric to circumferential and is associated with an almost complete loss of the intestinal layering (arrows). (E) At 9 weeks, the jejunal mass changes to an ill-defined mixture of hypoechoic and hyperechoic areas with circumferential transmural mass growth (full thickness: 1.4 cm) and complete wall layering loss

Serial ultrasonographic examinations (Aplio i800; Cannon Medical System, Tochigi, Japan) were performed over 18 weeks, at intervals up to three weeks, from initial presentation until death (Figure 3). Initially, the abdominal ultrasound showed diffuse and generalised jejunal wall thickening, particularly in the muscularis layer, with normal wall layering (Figure 3A). The jejunal and ileocecal lymph nodes were normal. Famotidine (Gaster Tab; Donga ST, Seoul, Republic of Korea; 0.5 mg/kg q12 h), maropitant (Cerenia; Fareva Amboise, Porce sur Cisse, France; 2 mg/kg q24 h), amoxicillin (Amocla Tab; Kuhnil, Seoul, Republic of Korea; 15 mg/kg q12 h), and prednisolone (Solondo Tab; Yuhan, Seoul, Republic of Korea; 0.5 mg/kg q12 h) were administered orally accompanied by dietary therapy, but there was no improvement in the jejunal thickening after one week. At the 3-week recheck, the ultrasound revealed a poorly marginated, heterogeneous, eccentric intramural mass (7 × 10 × 11 mm) with altered wall layers in the thickest jejunal segment with peripheral mesenteric hyperechoic changes (Figure 3B). Perinodal hyperechoic changes of the ileocecal and jejunal lymph nodes were also noted. The dosage of prednisolone was increased to 1 mg/kg q12 h. Full-thickness, punch biopsies of the jejunal mass, one jejunal segment, and jejunal lymph node were performed. Five weeks after the initial presentation, corresponding to 2 weeks after the biopsy, a suspected stromal tumour with fibrosis, inflammatory bowel disease (IBD), and paracortical lymph node hyperplasia was revealed by the histopathological examination. The jejunal mass revealed features indicative of a mesenchymal-origin tumour likely to be a stromal tumour. It exhibited invasive growth within the muscular layer, accompanied by oedema and inflammation. Tumour cells appeared round to polygonal with hypertrophic nuclei, eosinophilic cytoplasm, and numerous vacuoles. The mitotic count was 5/10 in high-power fields. Due to a discrepancy with the diagnostic imaging findings, additional immunohistochemical staining for c-Kit was referred to confirm the stromal tumour. At this point, prednisolone began to be administered at an increased dosage of 1.25 mg/kg q12 h. Seven weeks after the initial presentation, positive c-Kit staining was confirmed. During the 9 weeks from the first presentation, the lesion suspected of a stromal tumour gradually changed to an ill-defined, circumferential transmural mass with a complete loss of wall layering, while other segments showed similar or slightly increased thickening (Figure 3C–E).

At 9 weeks, the jejunal mass was surgically removed, and FGESF was diagnosed which differed from the biopsy results. On histopathology, the jejunal mass revealed widespread collagenous fibres with prominent sclerosis. Lesions extended from the submucosa to the serosal layer, with deep infiltration of eosinophils. Under high-power examination, the fibrous tissue showed an anastomosing pattern, hypertrophic myofibroblasts, and prominent eosinophil infiltration. Additional immunohistochemical staining for c-Kit was performed, and the negative c-Kit staining ruled out a stromal tumour. The prednisolone dosage was increased to 1.5 mg/kg q12 h.

The follow-up ultrasounds were conducted during the 9 weeks following surgery at 1- to 10-day intervals. Recurrence was not observed at the anastomosis site; however, persistent wall thickening continued, as well as in other segments of the jejunum. Progressively enlarged ileocecal and jejunal lymph nodes appeared 3 weeks post-surgery. Diarrhoea was detected 8 days after surgery and a faecal conventional microscopic examination, polymerase chain reaction (PCR) assay, and bacterial culture were performed. In the conventional microscopic examination, bacterial overgrowth and *Proteus mirabilis*, *E.* *coli*, and *Enterococcus gallinarum* were detected in the bacterial culture while the PCR was negative for 16 pathogens, including *Campylobacter coli*, *Campylobacter jejuni*, Feline parvovirus, *Tritrichomonas foetus*, Feline leukaemia virus, *Giardia lamblia*, *Toxocara cati*, *Toxoplasma gondii*, *Cryptococcus* spp., *Cryptosporidium* spp., Feline coronavirus, *Salmonella* spp., and *E. coli* spp. representing enterohemorrhagic, enteroinvasive, enteropathogenic, and enterotoxigenic pathogens. Additional antibiotics, including marbofloxacin (Marbocyl; Vetoquinol, Lure, France; 3 mg/kg q24 h) and metronidazole (Flasinyl tab; inno. N, Cheongju, Republic of Korea; 15 mg/kg q12 h) with mirtazapine (Remeron; Organon Inc, London, UK; 1.88 mg q12 h), were additionally prescribed. Despite intensive medical therapy, the patient exhibited progressive deterioration. Chlorambucil (Leukeran, Aspen, Switzerland; 15 mg/m^2^ q24 h) was also additionally prescribed 9 weeks post-operation in an attempt to manage the FGESF; however, the patient died 9 weeks post-operation due to unresponsive diarrhoea and anorexia, leading to cachexia. No post-mortem examination was conducted.

## DISCUSSION AND CONCLUSIONS

FGESF is predominantly observed in middle-aged adult cats, with a median age of 7–8 years (Craig et al. 2009; Linton et al. 2015). Although genetics, notably in male Ragdoll cats, and possible infections are observed, the aetiology remains uncertain (Craig et al. 2009; Grau-Roma et al. 2014; Linton et al. 2015; Martineau et al. 2023). Clinical signs include vomiting, diarrhoea, weight loss, and lethargy (Craig et al. 2009; Weissman et al. 2013; Linton et al. 2015), with over half of the affected cats presenting with peripheral eosinophilia (Craig et al. 2009; Linton et al. 2015). In fine needle aspiration (FNA) cytology tests of the masses, most cats show either non-diagnostic results, necrosis, or mixed inflammation, while less than half of the cases show eosinophilic inflammation. The FNA results of the lymph nodes are usually non-diagnostic or indicate reactive lymphadenopathy, with only a few cases showing eosinophils (Cerna et al. 2024). While case 1 shares many characteristics with previous FGESF cases, such as the presence of a pyloric lesion in a male Ragdoll cat with few eosinophils found in the cytology tests of the lymph node, it is notable that the clinical signs manifested at a much younger age compared to previous cases. Distinguishing FGESF from other conditions was challenging due to the young age, lack of peripheral eosinophilia, and diagnostic imaging features. This highlights the importance of a histopathological examination, and FGESF should be considered as a differential diagnosis in cats with pyloric masses, even in kittens.

In most FGESF case studies, FGESF presents as a single, heterogeneous, intramural mass with wall layering loss and regional lymphadenopathy on ultrasound, which resembles intestinal neoplastic lesions (Penninck et al. 1994; Weissman et al. 2013; Linton et al. 2015; Simeoni et al. 2020). Occasionally, FGESF exhibits a hyperechoic area, indicative of fibrotic regions, typically unobserved in neoplasia (Weissman et al. 2013; Linton et al. 2015). In case 1, the overall morphology of the pyloric mass and regional lymphadenopathy corresponded to previous reports, except for the protruding mass extending into the gastric lumen. In case 2, muscularis layer thickening of the jejunum was initially observed; however, it gradually progressed into a small eccentric jejunal mass with partial wall layering loss and finally progressed to a circumferential mass with complete wall layering loss. The lymph nodes changed from a normal structure to perinodal hyperechoic changes, followed by marked lymphadenopathy. Similar to this, in a previous FGESF case, only thickening of the intestinal wall without wall layering loss was noted at first presentation, later progressing to a mass that is refractory to medications (Martineau et al. 2023). This suggests that the possibility of an early state of FGESF should be considered in cases of intestinal wall thickening with poor response to medication. When considering that most of the previous reports described imaging features of FGESF in the advanced state (Weissman et al. 2013; Linton et al. 2015), these findings would be helpful in the diagnosis of early stage FGESF in veterinary practice.

In case 2, FGESF initially appeared in the thickest jejunal segment on ultrasound, whereas other segments had IBD. The exact aetiology and progression of FGESF remain unclear, despite its suggested association with immunological dysregulation (Linton et al. 2015; Martineau et al. 2023). In humans with IBD, inflammatory infiltrates promote phenotypic changes in fibroblasts through enhancement of TGF-β1 and IGF-1 expression, thereby altering the type III : I collagen ratio, leading to intestinal fibrosis. However, this procedure has nonspecific consequences for inflammatory cell infiltrates (Lawrance et al. 2001). It differs from FGESF in that fibrogenic mediators of eosinophils, particularly major basic proteins, play a significant role in inducing fibrosis (Craig et al. 2009). However, in case 2, eosinophils were not the predominant inflammatory cells on the first biopsy, while a mass with fibrosis was identified. Additionally, a recent immunohistochemical study on FGESF showed TGF-β1 playing a major role (Porras et al. 2022). These findings suggest a potential connection between IBD and FGESF, warranting further investigation.

Although CT serves as a valuable imaging tool for diagnosing and staging gastrointestinal tumours, there is a paucity of studies in cats (Simeoni et al. 2020; Tanaka et al. 2022). Similarly, research on CT studies of FGESF remains limited, with only two reported cases of FGESF located in the extramural retroperitoneal area and rectum (Lawrance et al. 2001; Thieme et al. 2019). In these two cases, FGESF showed heterogeneous contrast enhancement on CT, in contrast with our case, which illustrated mild homogeneous contrast enhancement (Thieme et al. 2019; Goffart et al. 2022). Considering the absence of necrotic lesions on the histological examination in case 1, the contrast enhancement pattern difference is likely attributable to the presence or absence of necrotic lesions. In a comparative CT study between lymphoma and adenocarcinoma in cats, adenocarcinoma demonstrated obstruction and layering enhancement on the post-contrast, whereas lymphoma showed lymphadenopathy (Tanaka et al. 2022). In case 1, pyloric obstruction, lymphadenopathy, and absence of layering contrast enhancement were observed, which overlapped with features of feline gastrointestinal lymphoma and adenocarcinoma. Thus, while CT is excellent for assessing lesion extent and adjacent lymphadenopathy, limitations in distinguishing between FGESF and gastrointestinal tumours exist. A histopathological examination should be performed in conjunction with CT.

There were several limitations in case 2. First, the nature of the ultrasound examination led to uncertainty in the evaluation of the same intestinal segment and the possibility that a small-sized mass was obscured by intraluminal gas. However, because the mass formed in the thickest jejunal segment and full tracking of the small bowel was performed in each examination, these possibilities seem less likely. Second, the initial biopsy may have functioned as an external factor that could have influenced the FGESF progression (Tai et al. 2021). Third, the initial biopsy resulted in a misdiagnosis of the mass as a gastrointestinal stromal tumour, causing confusion in communication with the owner regarding the treatment direction and delaying any appropriate treatment initiation. The misdiagnosis, particularly the discrepancy of the c-Kit results between the biopsy and post-surgical histopathological examination of the entire mass, may be due to the difference in the sampling methods or the interaction of c-Kit positive cells, such as the interstitial cells of Cajal in the gastrointestinal tract, with stem cell factor secreted by fibroblasts. This interaction could promote eosinophil adherence and initiate stimuli for eosinophil migration into inflamed tissues (Oliveira et al. 2002). Consequently, although the c-Kit was initially positive in the early FGESF, c-Kit results may become negative as fibrosis and inflammation progress (Kaji and Hori 2023).

We presented two cases of FGESF in the pyloric and jejunal regions and the diagnostic imaging features of ultrasound and CT. In both the ultrasound and CT, its appearance resembles that of gastrointestinal neoplastic lesions. On ultrasound, early FGESF on the jejunum may only show muscularis layer thickening. FGESF should be considered as a differential diagnosis for a pyloric mass with wall layering loss and lymphadenopathy, even in kittens. Serial ultrasound and biopsy should be conducted to confirm the presence of FGESF in cases of intestinal muscularis thickening unresponsive to medication.
